# Supplementary material for: A network analysis of problematic smartphone use in Japanese young adults
Source: PLoS One. 2022 Aug 8;17(8):e0272803. doi: 10.1371/journal.pone.0272803 (PMC9359578; doi:10.1371/journal.pone.0272803)
Supplement: S1 File — SAS-SV: Smartphone Addiction Scale–Short Version. (DOCX) [file pone.0272803.s001.docx]

**Supporting Information 1: Edge weights in the Smartphone Addiction network in the study participants**

| **Connected nodes** | | **Edge weights** |
| --- | --- | --- |
| SAS-SV1 | SAS-SV2 | 0.49 |
| SAS-SV1 | SAS-SV3 | 0 |
| SAS-SV1 | SAS-SV4 | 0 |
| SAS-SV1 | SAS-SV5 | 0 |
| SAS-SV1 | SAS-SV6 | 0 |
| SAS-SV1 | SAS-SV7 | 0.03 |
| SAS-SV1 | SAS-SV8 | 0 |
| SAS-SV1 | SAS-SV9 | 0.16 |
| SAS-SV1 | SAS-SV10 | 0.08 |
| SAS-SV2 | SAS-SV3 | 0.11 |
| SAS-SV2 | SAS-SV4 | 0.05 |
| SAS-SV2 | SAS-SV5 | 0.05 |
| SAS-SV2 | SAS-SV6 | 0.01 |
| SAS-SV2 | SAS-SV7 | 0.08 |
| SAS-SV2 | SAS-SV8 | 0.03 |
| SAS-SV2 | SAS-SV9 | 0.16 |
| SAS-SV2 | SAS-SV10 | 0 |
| SAS-SV3 | SAS-SV4 | 0.02 |
| SAS-SV3 | SAS-SV5 | 0.09 |
| SAS-SV3 | SAS-SV6 | 0.09 |
| SAS-SV3 | SAS-SV7 | 0 |
| SAS-SV3 | SAS-SV8 | 0.01 |
| SAS-SV3 | SAS-SV9 | 0 |
| SAS-SV3 | SAS-SV10 | 0.09 |
| SAS-SV4 | SAS-SV5 | 0.27 |
| SAS-SV4 | SAS-SV6 | 0.09 |
| SAS-SV4 | SAS-SV7 | 0.33 |
| SAS-SV4 | SAS-SV8 | 0.07 |
| SAS-SV4 | SAS-SV9 | 0.02 |
| SAS-SV4 | SAS-SV10 | 0.08 |
| SAS-SV5 | SAS-SV6 | 0.41 |
| SAS-SV5 | SAS-SV7 | 0.05 |
| SAS-SV5 | SAS-SV8 | 0.16 |
| SAS-SV5 | SAS-SV9 | 0 |
| SAS-SV5 | SAS-SV10 | 0.04 |
| SAS-SV6 | SAS-SV7 | 0.07 |
| SAS-SV6 | SAS-SV8 | 0.08 |
| SAS-SV6 | SAS-SV9 | 0 |
| SAS-SV6 | SAS-SV10 | 0.21 |
| SAS-SV7 | SAS-SV8 | 0.10 |
| SAS-SV7 | SAS-SV9 | 0.18 |
| SAS-SV7 | SAS-SV10 | 0.03 |
| SAS-SV8 | SAS-SV9 | 0 |
| SAS-SV8 | SAS-SV10 | 0.08 |
| SAS-SV9 | SAS-SV10 | 0.16 |
|  |  |  |

SAS-SV: Smartphone Addiction Scale – Short Version
