# Supplementary material for: A network analysis of problematic smartphone use in Japanese young adults
Source: PLoS One. 2022 Aug 8;17(8):e0272803. doi: 10.1371/journal.pone.0272803 (PMC9359578; doi:10.1371/journal.pone.0272803)
Supplement: S2 File — The panel above presents edge weights of the estimated network of 10 Smartphone Addiction Scale items and 95% confidence intervals (CIs) calculated using bootstrapping. The red line represents the original sample values, the black dots represent the bootstrap means, and the gray areas indicate the bootstrapped CIs. Each horizontal line represents one edge of the network, ordered from the edge with the highest edge-weight to the edge with the lowest edge-weight. The y-axis labels were removed to avoid cluttering for the simplicity of the graph. Figure indicates that many edge-weights likely do not significantly differ from one-another. The generally large bootstrapped CIs imply that interpreting the order of most edges in the network should be done with care. (DOCX) [file pone.0272803.s002.docx]

**S2:** **Bootstrapped 95% confidence intervals of edge weights**

The panel above presents edge weights of the estimated network of 10 Smartphone Addiction Scale items and 95% confidence intervals (CIs) calculated using bootstrapping. The red line represents the original sample values, the black dots represent the bootstrap means, and the gray areas indicate the bootstrapped CIs. Each horizontal line represents one edge of the network, ordered from the edge with the highest edge-weight to the edge with the lowest edge-weight. The y-axis labels were removed to avoid cluttering for the simplicity of the graph.

Figure indicates that many edge-weights likely do not significantly differ from one-another. The generally large bootstrapped CIs imply that interpreting the order of most edges in the network should be done with care.
