# Supplementary material for: A network analysis of problematic smartphone use in Japanese young adults
Source: PLoS One. 2022 Aug 8;17(8):e0272803. doi: 10.1371/journal.pone.0272803 (PMC9359578; doi:10.1371/journal.pone.0272803)
Supplement: S3 File — In this graph, each point on the x and y axes represents a pair of edges identified in a given network. Black boxes indicate significant differences between two edges (a bootstrap stability difference test: alpha = 0.05), whereas gray boxes do not indicate any significant differences. The diagonal represents the edge strength, where white indicates weak edge strengths, while blue indicates strong edge strengths. (DOCX) [file pone.0272803.s003.docx]

**S3: Edge difference test**

In this graph, each point on the x and y axes represents a pair of edges identified in a given network. Black boxes indicate significant differences between two edges (a boostrap stability difference test: alpha = 0.05), whereas gray boxes do not indicate any significant differences. The diagonal represents the edge strength, where white indicates weak edge strengths, while blue indicates strong edge strengths.
