# Supplementary material for: A network analysis of problematic smartphone use in Japanese young adults
Source: PLoS One. 2022 Aug 8;17(8):e0272803. doi: 10.1371/journal.pone.0272803 (PMC9359578; doi:10.1371/journal.pone.0272803)
Supplement: S4 File — The figure above presents the centrality stability as assessed using the case-dropping bootstrap method. Stability was assessed by re-estimating the network based on increasingly smaller subsets of the original sample. (DOCX) [file pone.0272803.s004.docx]

**S4: Stability of network centrality**

The figure above presents the centrality stability as assessed using the case-dropping bootstrap method. Stability was assessed by re-estimating the network based on increasingly smaller subsets of the original sample.
