# Supplementary material for: A network analysis of problematic smartphone use in Japanese young adults
Source: PLoS One. 2022 Aug 8;17(8):e0272803. doi: 10.1371/journal.pone.0272803 (PMC9359578; doi:10.1371/journal.pone.0272803)
Supplement: S5 File — (DOCX) [file pone.0272803.s005.docx]

**Supporting information 5: R code used for data analysis in the present study**

#package install

install.packages("ltm")

library("ltm")

install.packages("qgraph")

library("qgraph")

install.packages("ggplot2")

library("ggplot2")

install.packages("bootnet")

library("bootnet")

smaphodata <- read.csv("smartphoneadd.csv")

smaphodata <- na.omit(smaphodata)

cronbach.alpha(smaphodata1[,-1])

#labels

longnames <- c("Miss planned work due to smartphone use",

"Difficulty focusing on assignments/work due to smartphone use",

"Feel pain in the wrists or the neck while using a smartphone",

"Unable to stand not carrying a smartphone",

"Feel impatient and uneasy when not holding my smartphone",

"Have my smartphone on my mind even when not using it",

"Continued use of my smartphone despite its negative impact

on daily life",

"Constantly check my smartphone not to miss information on SNS",

"Use my smartphone longer than I intend",

"People tell me that I use my smartphone too much")

shortnames <- c("SA1",

"SA2",

"SA3",

"SA4",

"SA5",

"SA6",

"SA7",

"SA8",

"SA9",

"SA10"

)

centralitynames <- c("SA1: Miss planned work due to smartphone use",

"SA2: Difficulty focusing on assignments/work due to smartphone use",

"SA3: Feel pain in the wrists or the neck while using a smartphone",

"SA4: Unable to stand not carrying a smartphone",

"SA5: Feel impatient and uneasy when not holding my smartphone",

"SA6: Have my smartphone on my mind even when not using it",

"SA7: Continued use of my smartphone despite its negative impact on daily life",

"SA8: Constantly check my smartphone not to miss information on SNS",

"SA9: Use my smartphone longer than I intend",

"SA10: People tell me that I use my smartphone too much")

#Regularized graph

net<- estimateNetwork(smaphodata, default="EBICglasso",

corMethod = "spearman")

pdf("smartphoneaddiction_network.pdf", paper = "A4r", width = 12, height = 7)

q <- qgraph(net$graph, layout = "spring",

labels = shortnames, nodeNames=longnames, legend=TRUE,vsize = 4,

title = "Smartphone Addiction Scale in college students",

legend.cex=0.4, GLratio = 1.3)

dev.off()

#centrality

centRes <- centrality(net)

pdf("centrality_smartphoneadd.pdf", paper = "A4r", width = 12, height = 7)

centralityPlot(net, include = c("Strength", "Betweenness", "Closeness"),

labels=centralitynames)

dev.off()

#accuracy

b <- bootnet(net, nBoots=1000, nCore = 8, default = "EBICglasso")

plot(b)

pdf("boot_smapho.pdf")

plot(b, labels = TRUE, order = "sample", plot = "difference", onlyNonZero = TRUE)

dev.off()

b1 <- bootnet(net, nBoots=1000, nCore = 8, default = "EBICglasso")

plot(b1)

pdf("boot_CI_smapho.pdf")

plot(b1, labels = FALSE, order = "sample")

dev.off()

b4 <- bootnet(net, nBoots=1000, statistics = c("Strength", "Betweenness","Closeness"), type = "case", nCore =8)

corStability(b4)

pdf("stability_centrality_smapho_network.pdf")

plot(b4, statistics = c("Strength", "Betweenness","Closeness"))

dev.off()
