# Supplementary material for: A network analysis of problematic smartphone use in Japanese young adults
Source: PLoS One. 2022 Aug 8;17(8):e0272803. doi: 10.1371/journal.pone.0272803 (PMC9359578; doi:10.1371/journal.pone.0272803)
Supplement: Attachment — Submitted filename: Authors reply to reviewers_PlosOne.docx [file pone.0272803.s007.docx]

**Our replies to reviewers’ comments**

Reviewer #1: In this article the authors perform an exploratory network analysis of the answers to the smartphone addiction scale-short version of 487 Japanese college and university students. This included estimating the network and 3 centrality indices. The authors also proceed to divide the sample according to gender and estimate two sub-sample networks, testing for their differences. They found that preoccupation and withdrawal related items are particularly important in the network and also found differences between male and female subsample networks.
The topic of the article is of great importance and the use of network analysis could be of significance to further improve the knowledge about addiction disorders. The network analysis methodology is overall well applied. The findings about the most central symptoms could indeed be of interest to the field.
However, this reviewer thinks the study’s novelty is limited and also that it lacks a robust discussion of the results.
The overall quality of the writing is modest.

Major issues

- Study’s novelty: the cited study by Andrade, 2020 also performs a network analysis of the same instrument as the current study. More so, it frames it in a validation context of the instrument, making it a sounder scientific effort than the current one. The authors claim however that the fact that they’re using a slightly older sample justifies the study’s novelty. That difference seems to this reviewer as a very small one (in the end the results did not differ much, proving that the difference was not so relevant).

**Our reply: Thank you for your comment and input. We acknowledge that the fact we focused on different age samples (transitional age youth) from ones in the extant study may not sound substantially novel. However, we would like to emphasize that understanding how this addictive behavior functions at different developmental age is important as the ownership of smartphones and degree of parental control and possibly school rules (for students who attend compulsory school, such as middle school and high school) can influence problematic smartphone behavior. We added a sentence and a reference below to state that maladaptive and excessive use of smartphones was identified more frequently in college students than middle and high school students in the revised manuscript to support our concept**.

Discussion 1^st^ paragraph lines 11 – 13: “In fact, the survey conducted in Japan exhibited more maladaptive and excessive use of smartphones in college students compared to middle and high school students [35]”

- Some questions about the use of this instrument (SAS-SV) should be clarified, in particular its validation and/or the validation of the original SAS instrument in Japan. Was that work done previously? In this reviewer’s perspective that would be a fundamental first step before engaging in a study like the present one (in the cited Brazilian study, a careful validation of the SAS-SV is performed alongside network analysis)

**Our reply: The original instrument (SAS) was validated in Japanese youths in another study, where the authors reported the Japanese-version of SAS demonstrated good reliability and validity (DOI:** [**10.1007/s11469-021-00594-z**](http://dx.doi.org/10.1007/s11469-021-00594-z)**). We added this statement in the revised manuscript. We acknowledge that the short-version (SAS-SV) has not validated in Japanese samples yet.**

**Lines 5-6 in Measures > Smartphone Addiction Scale-Short-version: “The Japanese-version of the SAS was developed, and its reliability and factor validity were assessed in 1,037 youth aged 15 – 24 years [28].”**

- Regarding the sample, it represents a group of students from a specific region of Japan and with high gender distribution imbalance. This limits generalizability as the authors point. However, a more detailed description of the sample and its recruitment is necessary. For instance: when was the data collected? In which schools, from which specific region? Was there a rational for choosing this sample? Could the authors explain better the setting in which the questionnaire was filled? Were there potential participants who did not answer to the questionnaire? Simultaneously, a better sociodemographic description of these individuals should be provided – detailed school level description, age range (min and max values), and ideally some information that could inform the reader about socioeconomic status of this sample.

**Our reply: We added descriptions of the study and study participants in the revised manuscript.**

**Participants subsection in the Material and methods section: “The subjects of the study were 487 private college and university students in Sapporo city and its environs in Japan. Study participation rate was 81.2% (487/600). Their academic deviation scores were average or a little below the average compared to the national standard in Japan. Research collaborators for data collection were recruited through personal connections of the first author of this paper (MT). Nine teachers from three universities and six colleges agreed to voluntarily support our investigation.”**

**We did not collect socioeconomic status or detailed characteristic information about participants except for that already reported in the manuscript.**

- Regarding the study’s aims, the reviewer believes the network comparison performed is lacking a justification. The authors should explain why they believe it is relevant to compare networks based on a gender dichotomization of the sample. Also, one problem regarding this aim is the imbalance on sub sample size. Given that the starting point is already relatively modest, the authors are left with small samples for the sub-analysis, particularly the men’s network. Was the stability of each of the subsample networks analyzed?

**Our reply: We agree with the reviewer’s point. We acknowledge that the comparison test was exploratory without sufficient scientific justification. Additionally, as pointed by the reviewer, even we conduct the network comparison test, the sample size differences between female and male students and the small sub-sample size of male students would result in skewed findings. Thus, the authors determined to omit this analysis and remove corresponding paragraphs from the manuscript.**

- As a general comment, the discussion section should be improved. In the current form it stresses too much aspects that are limited by design in the current study (eg ideas about how addiction disorders are formed or should be treated).

**Our reply:** **Thank you so much for your comments. We revised our manuscript, including discussion based on reviewers’ feedback.**

- The writing should be improved. A native English speaker with experience in scientific writing could help improve substantially the article (see in minor issues some passages to reconsider).

**Our reply: We asked a native English speaker who holds a doctor’s degree and is an acquaintance of one of the authors (MT) to proofread the manuscript.**

- A justification for not providing the data should be given (PLoS Data policy); This reviewer also strongly encourages the authors to make data analysis information available as supplementary material (R code)

**Our reply: We uploaded the R code use for the analysis in this study in Supplementary data 5.**

Minor issues

- Smartphone addiction concept: the authors point the lack of consensus around this construct. The reviewer would ask the authors to consider the use of ‘Problematic Smartphone Use’ instead

**Our reply: We agree with the authors’ suggestion. We changed the term “smartphone addiction” to “problematic smartphone” throughout the manuscript.**

- On a more technical note about the network comparison performed, the authors determine that they will perform 3 different tests in their methods section. As van Borkulo et al point out in their paper explaining this procedure, the edge strength invariance test should only be performed if there is an a priori hypothesis about specific edges to directly test between the networks, or, in case there is no a priori hypothesis, it could be done as a follow-up to a significant difference in the structural invariance test. Therefore, in this reviewer’s opinion, since there is no a priori hypothesis for the authors, in the methods section it should be specified that this test (edge strength invariance) will only be performed if a difference in structural invariance is found. Then, in the results section, the network comparison tests report seems to be a bit unclear. The authors state that there was a “significant difference for the overall network structure” but explain this as meaning that the “nodes were more densely connected overall in female students than in male students”. This explanation refers to the invariant global strength aspect of the test and not the invariant network structure. So, this reviewer is confused about what is being reported – invariant network structure or invariant global strength? The edge weight comparisons reported afterwards should only be pursued in case network structure differs. (See: Van Borkulo, C. D., Boschloo, L., Kossakowski, J., Tio, P., Schoevers, R. A., Borsboom, D., & Waldorp, L. J. (2017). Comparing network structures on three aspects: A permutation test. )

**Our reply: Thank you for suggested papers and your constructive comments. We agree with the reviewer’s points and concerns. In the revised manuscript, we determined not to conduct the network comparison test given no priori hypotheses driving this analysis and as the study findings from this analysis could be skewed and may lack the accuracy due to the small sub-sample size of male students and inequality of sample size between female and male students.**

- The meaning of centrality indices should be rectified. On page 13 line 4, the way strength centrality is presented is not correct (ie, strength centrality does not translate the association of a node with “all other other nodes in the network” as stated. It is instead a measure of local influence). The meaning of betweenness and closeness centrality measures on cross-sectional data network analysis in the context of mental disorders has also been questioned. I would suggest the author to check references such as the following for this matter: Bringmann, L. F., Elmer, T., Epskamp, S., Krause, R. W., Schoch, D., Wichers, M., ... & Snippe, E. (2019). What do centrality measures measure in psychological networks?. Journal of abnormal psychology, 128(8), 892.

**Our reply: The authors very much appreciate the reviewer’s feedback and suggested papers. We addressed this point in the revised manuscript (see the “Centrality” subsection in the “Material and methods” section).**

- In the methods section it should be mentioned which option was chosen for the network layout.

**Our reply: We used the Fruchterman-Reingold algorithm, which we added to the revised manuscript (the last sentence in the network estimation subsection under the Data analyses section).**

- Table 1: the asterisks in the Age and SAS-SV lines are a bit confusing and seem unnecessary to this reviewer; the readability of the table could be improved if the “(mean±SD” parts were positioned without a line break and if some hierarchical visual cue could be added to subdivisions of “internet use (hrs) and “purpose”; the acronym SNS should be explained in the table legend.

**Our reply: All addressed in the revised manuscript.**

- Network estimation subsection from analytic plans: please use proper reference for qgraph package. A reference for the R statistical software is also lacking.

**Our reply: The reference is now placed in the revised manuscript (#30). Thank you.**

- Network estimation subsection from analytic plans: the sentence “leading to a sparse network with explanatory power”. What is meant by 'explanatory power'?

**Our reply: We decided not to add the phrase pointed by the reviewer as the statement was vague and confusing.**

- Results, network structure and centrality, paragraph2: “The weights of the edges between the items SA1 (…) and SA2 (…) and the items SA5 (…) were stronger than others” – it is unclear which 2 pairs of nodes are the authors referring too. Also, the use of “stronger than others” in this context seems imprecise as this is not being tested.

**Our reply: Thank you for pointing this out. To clarify our intents, we revised the manuscript as follows (changes are highlighted in yellow): “The weights of the edges between the items SA1 (Miss planned work due to smartphone use) and SA2 (Difficulty focusing on assignments/work due to smartphone use) and those between the items SA5 (Feel impatient and uneasy when not holding my smartphone) were larger than others in the given network (0.49 and 0.41, respectively).”**

- Results, network structure and centrality, paragraph3: “Inspecting the network structure” – the observations in this sentence do not follow from “inspection” of the network structure but instead of the centrality values. The visual layout of the network is rather arbitrary.

**Our reply: We agree with the reviewer’s point. We deleted that phrase in the revised manuscript. Corresponding sentence in the revised manuscript is below: “SA6 (Have my smartphone on my mind even when not using it) was with the highest closeness and relatively high strength in centrality indices.”**

- Results, network structure and centrality, paragraph3: “strongly associated with SA5, the central node” please rephrase. The qualification “the central node” is imprecise. SA5 is the node with highest strength centrality.

**Our reply: Thank you for this feedback. We corrected the corresponding sentence as follows in the revised manuscript.**

**“In particular, while this item was strongly associated with SA5, it also had the moderate association with SA10 (People around me tell me that I use my smartphone too much) that was only weakly associated** **with the node with the highest strength centrality (SA5).”**

- Results, network structure and centrality, paragraph3: “moderate association with SA9”. It seems that SA10 is meant here instead of SA9.

**Our reply: It was a typo. The reviewer was right. We corrected this error in the revised manuscript.**

- Discussion paragraph 1: "findings from the present study could further our understanding of how smartphone addiction psychopathology is developed and maintained" - please reconsider. Cross-sectional network analysis cannot inform about dynamic aspects of mental disorders such as their development or maintenance.

- Discussion, paragraph 4: please consider rephrasing how you draw conclusions from your study to therapeutic approaches. It seems that the current formulation is too strong given the limitations of the current study design and implementation.

- Discussion, paragraph 4: There is a long description of current approaches to treat behavioral addictions. This is however disconnected from the current study’s results (and purposes) and therefore seems misplaced in the discussion.

- Discussion, paragraph 5: The explanation given about the differences between male and female subsample networks is not very sound in the opinion of this reviewer. It is unclear how “different psychological backgrounds” (a concept that is not very specific) would lead to “strong edge weights”. The remark about a supposed female ‘fear of missing out’ seems arbitrarily used. It should be discussed however if the differences in connectivity found could be attributed in the first place to female higher scores and a ‘floor-effect’ generated by lower scores on the male side.

- Conclusions: the use of “formation” of psychopathology is inaccurate because a cross-sectional network cannot inform about causality or dynamic patterns related to how psychopathology emerges.

- Conclusions: the sentence “Findings from this network analysis would provide us with deeper understandings of smartphone addiction” is too vague. In the end the reader struggles to find this study's take-away messages.

- English writing aspects – the reviewer suggests that the text is reviewed by a native English speaker. Here are however some suggestions from the reviewer:
-Introduction, second paragraph: the reviewer would suggest the authors to remove the sentence “The superb mobility and multifunction capability allows us to access the internet anytime and anywhere.
-Introduction, paragraph 3: “smartphone addiction is considered [A] behavioral addiction (A is lacking); “which a person is forced to engage – the use of “forced” seems too strong, please use a more adequate alternative;
-Introduction, paragraph 5: “including THE smartphone addiction scale” (THE is lacking)
-Introduction, paragraph 6: “behavioral addiction is complex human behaviors” should become “behavioral addiction is A complex human BEHAVIOR”
-Introduction, paragraph 7: “Rather, it is considered a ring as a complex network of mutually reinforcing symptoms” – this sentence is not clear, please rephrase, and consider changing the word “ring”.
-The section termed “analytic plans” should in this reviewer's view be renamed. Consider alternatives like “data analyses” or use “network analysis” as an umbrella that you then subdivide into subanalyses.
-Network estimation subsection: “represent individual signs (symptoms)”, consider using the term "item” as in this case the nodes represent items from an instrument.
-Centrality subsection: “sum of distances from the node to all other nodes):” use ";" instead of ":" here
-Centrality subsection: “we interpreted a symptom with the strongest strength as a central symptom in a given network” please rephrase
-Discussion, paragraph 1: “identified the important symptoms “. Rephrase paying attention to the fact that the use of THE important symptoms is inaccurate.
-Discussion, paragraph 3: “Huang and his colleagues conducted a network analysis of problematic smartphone use in grade 4 and 8 students in China using a self-rating scale, smartphone addiction proneness scale” – rewrite the end of the sentence
-“central indices” should be corrected to “centrality indices”

**Our reply: Thank you so much for these suggestions. We revised our manuscript based on these suggestions (corrected parts are highlighted in yellow in the revised manuscript). In addition, as stated above in our letter, we asked a native English speaker to proofread the revised manuscript.**

Reviewer #2: It is my pleasure to read this study with adequate sample and exact analysis. I have some suggestion for improving the manuscript.

1 Please include the theory or evidence for the addiction characteristics of excessive smart phone use in the introduction section.

**Our reply: As pointed out by the other reviewer, due to the lack of consensus on the constructs of smartphone addiction and no compelling theory supporting the concept of smartphone addiction at this point, we decided to use “problematic smartphone use” instead throughout the revised manuscript.**

2 The female sample is larger than male. Please explain the reason and detail for possible limitation, especially for the gender effect on smartphone use.

**Our reply: As answered above to respond to the other reviewer, we determined not to conduct the network comparison test as the study findings from this analysis could be skewed and may lack the accuracy due to the small sub-sample size of male students and inequality of sample size between female and male students.**

3 Since there is only ten item in the scale, please discuss whether the number of item will limit the analysis for the items.

**Our reply: The findings from network analysis depend on what items are entered in analysis; however, this does not mean the number of items in the scale influence accuracy and stability of network analysis findings.**

4 Pleas explain the result of the figure 1 in the revsied manuscript

**Our reply: Thank you for this comment. Figure 1 is only for visualization of network analytic findings. As discussed in other network analysis literature, it is recommended we discuss findings obtained from centrality analyses (figure 2) as visualization per se may be arbitrary. General description about network visualization is placed as a figure legend “Figure 1. Regularized partial correlation network of Smartphone Addiction Scale Short-Version in college and university students in Japan (N = 478). Green lines indicate a positive association. Line thickness reflects the strength of association, controlling for all other symptom nodes in the network.”**

5 Although s4 and s5 are the central of the network, however, the score in these two item was relative lower. Please explain it.

**Our reply: Strength centrality is a measure that reflects the degree to which each node is connected to other nodes in the network. Thus, the degree of centrality differs from the degree of item score.**

6 The author had well demonstraed the implication of the result of the study. I agree with most of their claim. However, this content was not proved in this study. Thus, it should prevent to provide this content as it had been proved. Other reference should be provide to support their claim.

**Our reply: Thank you for this comment. We agree with the reviewer’s point that the paragraph pertaining to the implication of the result of the study was not stated based on scientifically sound hypotheses. To avoid any confusion and to make the flow of the manuscript more coherent, we deleted this paragraph in the revised manuscript.**
