# Supplementary material for: A network analysis of problematic smartphone use in Japanese young adults
Source: PLoS One. 2022 Aug 8;17(8):e0272803. doi: 10.1371/journal.pone.0272803 (PMC9359578; doi:10.1371/journal.pone.0272803)
Supplement: Attachment — Submitted filename: Response to the reviewer_PLOSONE_R2.docx [file pone.0272803.s008.docx]

**Response to the reviewer #1 (Dr. Bernardo Melo Moura)**

Reviewer #1: The authors conducted several changes in the manuscript that contributed to a much stronger work. Namely, the writing quality is now much improved, the analyses performed and their justification became clearer, and the availability of data and code now made this a transparent and potentially replicable scientific work.

**Our response: Thank you so much for your positive feedback. We truly appreciate your constructive comments on the previous manuscript, which improved the quality of this manuscript.**

There are some aspects though that I believe are still in need of revision in order for the manuscript to be acceptable for publication.

One general point is that, now that some unnecessary remarks were removed from the discussion, this section should be improved by adding thoughts about some of the issues the manuscript raises (see suggestions below).

(I acknowledge that some of my current minor points address issues already present in the first draft of the manuscript. Given the very high amount of changes required for the first revision, these points seemed less relevant then and/or are now in more clear need of revision given the other changes made to the article.)

Major issues

- In the abstract it is still mentioned that gender differences were explored in the network. This should be removed as that analysis is no longer performed.

**Our response: Thank you. That part is now removed.**

- Table 1 has separate columns for male and female participants. Given that there is no longer a network comparison according to gender, could the authors state why this is still relevant? I would also like to know the authors’ opinion about 2 further aspects related to this, that might be relevant for the discussion:

**Our response: We agree with the reviewer’s point. As we decided not to conduct network comparison analysis between female and male students due to imbalanced sample sizes, listing sample characteristics separately for female and male students does not make any sense in the manuscript. Therefore, we decided to list the sample characteristics of the whole participants alone in Table 1 and abbreviated the description about the participants’ characteristics in the manuscript in the Result section.**

1 ) why is it that ¾ of the sample are females (Is this something that mirrors the high-school/university demographics from Japan or is it a selection bias?) and how could that imbalance bias the results?

**Our response: This is purely due to a selection bias. The limitation stemming from this bias is mentioned in the discussion as below: “Regarding our sampling method, we recruited study participants only from colleges in one region in Japan, and the gender ratio was far from even, affecting the generalizability of our study findings.”**

2) how could the different smartphone usages (reported in table 1) affect the results (e.g. can we consider this study as mostly representing social network use disorder? Could different usages lead to different network structures hypothetically? Would this factor be more important than gender itself?). I believe these aspects are very important to be considered in the discussion and would enrich it very much.

**Our response: Thank you for your thoughtful comment. We added the following paragraph regarding this topic to the discussion (after the third paragraph) in the revised manuscript: “In the present study, more than half of the participants reported SNS as their primary use of the smartphone (62.2%), the percentage of which was higher than that of other reasons (gaming, for example). This raises a potential hypothesis that different smartphone usages lead to different network structures. Future studies with larger sample sizes would allow researchers to examine if the smartphone usage is an important factor contributing to network structures of problematic smartphone use.”**

Minor issues

- Abstract (and results too): I believe it is confusing to state “The estimated network yielded 45 edges, among which 34 edges had non- zero weights”. What are the 0 weight edges? Maybe just state that you’ve found 34 edges.

**Our response: Agreed and revised the manuscript accordingly.**

- Intro (last sentence of the 2nd to last paragraph) “population would not arguably be influenced by family”. I suggest that you consider stating something like “would not be directly influenced by family”, given that how a family raised the subject would continue to influence him/her afterwards.

**Our response: Agreed and revised the manuscript accordingly.**

- Intro (last paragraph): Second aim “identify which items could play important roles” – to play an important role is already an extrapolation. What we can do is to identify the most central symptoms in the network (hence, as you say before, the symptoms that potentially play a critical role in the onset and maintenance of the disorder). Consider rephrasing.

**Our response: thank you for this comment. We rephrased that part as “identify the most central symptoms in the network”.**

- Table 1 – consider reducing the decimal places to 3, but check exactly the journal guidelines on this. P-values less than 0.001 are usually reported as p<0.001.

**Our response: In the revised manuscript, we decided not to list characteristics of female and male students separately.**

- Is there some information available about the nearly 200 students who did not answer the questionnaire? For instance, about gender (were they predominantly male? this could explain your sample bias).

**Our response: No, unfortunately, we do not have any information about those students who decided not to answer the questionnaire.**

- Methods: citation for the qgraph package should be added (in data analyses - network estimation) <https://cran.r-project.org/web/packages/qgraph/citation.html#:~:text=qgraph%20citation%20info,4)%2C%201%E2%80%9318>.

**Our response: thank you. We added this as the citation #31.**

- Methods: please consider adding succinctly what the Fruchterman-Reingold algorithm does in terms of network layout (readers not acquainted with network methods might find it intriguing)

**Our response: We aded a brief explanation as below:**

**“a force-directed algorithm that encourages closely related nodes to be plotted near each other.”**

- Data analyses->centrality (first sentence): “We calculated several indices” – please consider rephrasing to “calculated three indices”

**Our response: rephrased as suggested.**

- Data analyses->centrality (second sentence): “a symptom with the highest value of strength was defined as a central symptom in a given network”. Consider rephrasing or removing this sentence as it is self-evident that the centrality is higher according to how high strength centrality is.

**Our response: thank you. We removed this phrase.**

- Data analyses->centrality (last sentence): “These are typically presented as standardized Z-scores”. Consider clarifying – “these” -> centrality values

**Our response: Revised as suggested. Thank you.**

- On the first subparagraph of the results section, the text repeats what can be already found in table 1. I would strongly recommend that the authors simply refer to the table for the information that can already be found there.

**Our response: This subparagraph is now simplified by eliminating detailed descriptions regarding the descriptive statistics as we decided not to list female and male students’ characteristics separately in Table 1.**

- Results -> network structure and stability (first sentence): “relations among SA symptoms”. I believe you dropped the SA terminology, so please consider changing it consistently in the manuscript. This includes changing the node names in the figures. For example, instead of using SA#, consider simply using the number 1-10 (as you do in table 2).

**Our response: We decided to consistently use the numbers SA1 – SA9 throughout the manuscript. Accordingly, we changed the numbers in Table 2 to SA1 – SA9.**

- Results -> network structure and stability (second paragraph, first sentence): “The weights of the edges between the items SA1 (…) were larger than others in the given network“. Please consider rephrasing as this does not seem to have been specifically tested (and you mentioned there is considerable overlap in confidence intervals).

**Our response: We decided to simply state that the weights of these edges were large in the revised manuscript.**

- Discussion: the term ‘smartphone addiction’ is sometimes used here in a way that is not completely harmonized within the manuscript given that there was an intention to substitute this term.

**Our response: We found that we used “smartphone addiction” twice in the following sentence in the previously submitted manuscript: “Although two recent studies examined the network structure of problematic smartphone use and smartphone addiction in school-age children in China [25] and Brazil [18], this is the first study that employed the network approach in understanding psychopathology of problematic smartphone use in college students in Japan who were transitioning to adulthood.”**

**The first one was intentionally used here to match the title of the citation (Andrade ALM, Scatena A, Martins GDG, Pinheiro BO, Becker da Silva A, et al. (2020) Validation of smartphone addiction scale - Short version (SAS-SV) in Brazilian adolescents. Addict Behav 110: 106540.). We rephrased the second one to “problematic smartphone use” to make the use of this terminology coherent throughout the manuscript.**

- Discussion (second paragraph, first sentence): “the Brazilian study” – please consider changing this. Would prefer either that you cite the authors name or that you refer to the “study conducted with [demographic group] in Brazil”

**Our response: revised as suggested.**

- Discussion (second paragraph, third sentence): “to produce [the] addiction cycle”

**Our response: Thank you. We revised the manuscript.**

- Discussion (second paragraph, fourth sentence): consider enriching the discussion by explaining how you believe overuse symptoms could still “contribute to either the development or maintenance of the network” even though your results do not seem to show them as central.

**Our response: Thank you for this comment. We added the following phrase (highlighted in yellow) to the corresponding sentence to enrich the discussion: “Although we did not identify strong centrality in items pertaining to continued overuse of the smartphone (SA9 and SA10 items), it is still possible that these symptoms contribute to either the development or maintenance of the network of problematic smartphone use via the associations between these overuse symptoms and preoccupation symptoms (edge weight 0.21 between SA6 and SA10, for example) based on the above-mentioned three-stage model of addiction”**

- Discussion (third paragraph, third sentence): “Differences in the symptom centrality between the study above and our study may be attributed to the difference in the scale used for each study.” Please consider enriching your discussion by stating your thoughts on why that is the case. What are the differences between the instruments?

**Our response: We added the following phrase to the corresponding sentence: “****as the functions of the scale modified by researchers in the above-mentioned study did not have withdrawal as one of the scale dimensions.”**

- Discussion (third paragraph, last sentence): “Thus, such smartphone users could have difficulties leaving their smartphones out of reach and feel anxious without the smartphone in close proximity.”. Good point. Consider making explicit that this is why you believe that an older population (like the one in your study) might have withdrawal symptoms as more central in the network than overuse symptoms.

**Our response: Thank you for your thoughtful suggestion. We added a sentence below at the end of this paragraph: “Accordingly, we believe that young adult population, compared to school age population, might have withdrawal symptoms as more central in the network than overuse symptoms in the present study.”**

- Figure 2 legend: “with higher scores indicative of greater importance within the overall network.” Same as stated above. Consider refraining from using the term “importance” as this is an extrapolation.

**Our response: We rephrased this part as “with higher scores indicative of greater influence within the overall network.”**
